# A case of classic neuromyelitis optica (Devic’s syndrome) triggered by pegylated-interferon α

**DOI:** 10.1186/2050-6511-15-56

**Published:** 2014-09-30

**Authors:** Davide Mangioni, Alessandro Soria, Laura Brighina, Alessandra Bandera, Carlo Ferrarese, Andrea Gori

**Affiliations:** 1 Division of Infectious Diseases, Department of Internal Medicine, San Gerardo Hospital, University of Milano-Bicocca, 20900 Monza, Italy; 2 Department of Neurology, San Gerardo Hospital, University of Milano-Bicocca, Monza, Italy

**Keywords:** Adverse events, HCV, Neuromyelitis optica, Pegylated-interferonα

## Abstract

**Background:**

Despite recent development of direct acting antivirals for treatment of hepatitis C, the current standard of care may still include pegylated-interferon, which is associated with frequent and, at times, serious adverse events.

**Case presentation:**

Here we report for the first time on a severe case of classic neuromyelitis optica (i.e.*,* optic-spinal form) in a 32 year-old Egyptian man with chronic hepatitis C treated with pegylated-interferon α2a for 4 months.

**Conclusions:**

Treating physicians must be alerted on rare but important unexpected complications of interferon, in order to consider carefully its use especially when they deal with patients not in dire need of urgent treatment.

## Background

Hepatitis C virus (HCV) chronically infects approximately 160 million people worldwide and ranks among the leading causes of liver disease. Even with recent licensing of first-generation protease inhibitors, pegylated-interferon α (Peg-IFNα) plus ribavirin remains the cornerstone of HCV treatment, especially in genotype 1 and 4 HCV infection [1]. Adverse events, including a number of neurological and autoimmune conditions, may be treatment-limiting and, rarely, life-threatening [2,3].

## Case presentation

**Figure 1:**
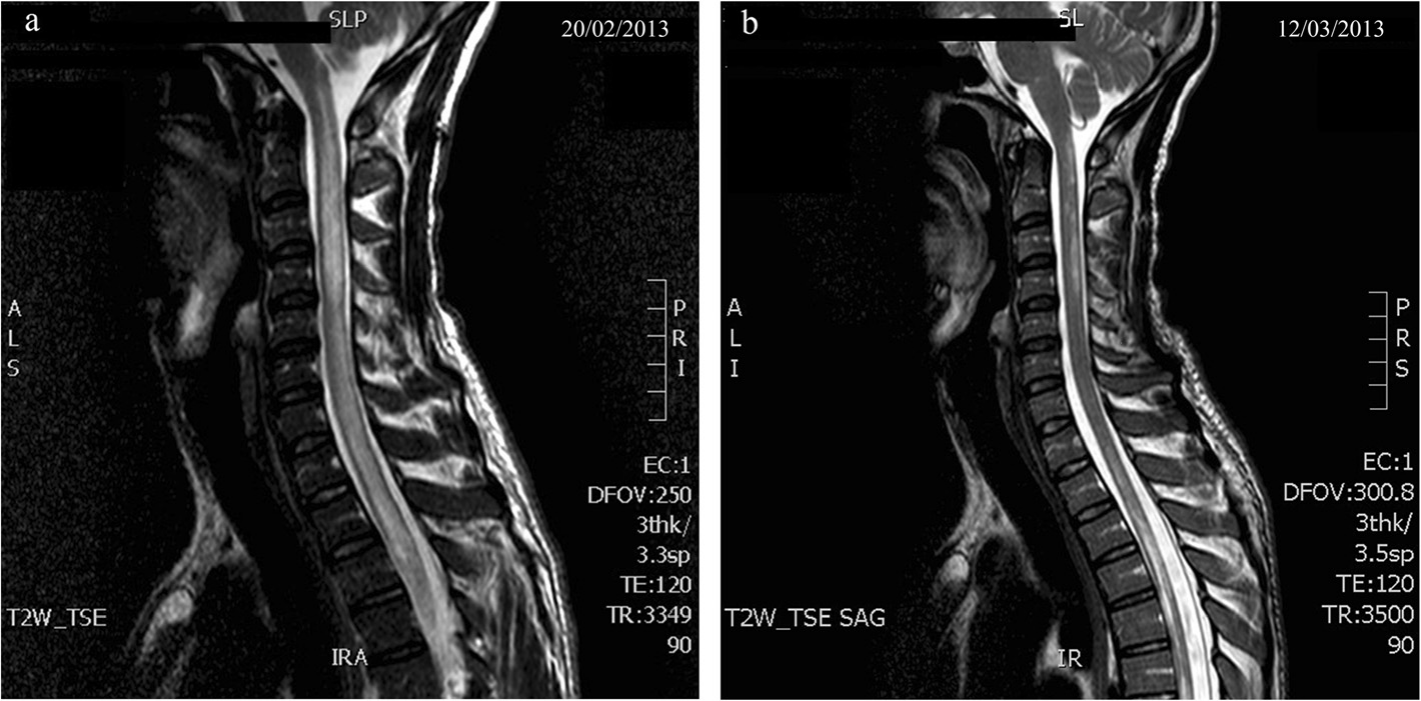
**Spinal cord MRI images before (a) and after (b) immunosuppressive treatment. a**: Sagittal spinal cord MRI image demonstrating signs of longitudinally extensive transverse myelitis that affects the entire spinal cord and spreads upwards to the lower regions of the medulla oblongata. **b**: Sagittal spinal cord MRI image which shows a clear reduction of the areas with altered signal intensity.

We report on a 32 year-old Egyptian man with chronic hepatitis C (genotype 4) diagnosed on July 2012. His medical history was otherwise unremarkable, particularly concerning neurologic or autoimmune disease. On October 2012, following liver biopsy (which showed mild liver disease: Ishak fibrosis 1) we began administering antiviral therapy with pegylated-interferon-α2a plus ribavirin, obtaining an early virological response with no significant side effects.In early February 2013, after 12 weeks of therapy, the patient complained about a rapid and progressive worsening of vision in his left eye. An urgent ophthalmologist examination diagnosed a retrobulbar optic neuritis, the symptoms of which spontaneously resolved a few days later. Two weeks after the onset of ocular symptoms, the patient returned to the emergency room for acute lower limb weakness accompanied by constipation and urinary retention. Neurological examination showed no abnormalities, except for a bilateral lower limb hyposthenia. Lumbar puncture revealed a moderate increase of the cerebrospinal fluid (CSF) proteins and normal CSF glucose, 15 leukocytes/μL (predominantly mononuclear), and absence of oligoclonal bands; CSF cultures and virological analyses were requested. As viral encephalitis was suspected, the patient was admitted to the Infectious Diseases ward, anti-HCV therapy was discontinued, and acyclovir was started. A few days later, neurological examination showed clear clinical deterioration, with flaccid paraplegia, constipation, hypoesthesia from D10 down (including the perineal region) and anesthesia below the knee. A spinal cord magnetic resonance imaging (MRI) demonstrated signs of longitudinally extensive transverse myelitis, affecting the entire spinal cord and spreading upwards to the lower regions of the medulla oblongata [Figure 1a].The patient was then transferred to the Neurology Department, as central nervous system (CNS) demyelinating disease was now suspected. Based on negative CSF cultures and virological analyses, acyclovir was stopped and intravenous corticosteroid boluses were initiated. To complete the diagnostic process we performed brain MRI, electromyography and blood screening panel for autoimmune diseases, all of which were normal. We also tested the presence of plasma Anti-aquaporin4 antibodies (AQP4-Ab) which turned out to be positive, confirming the diagnosis of neuromyelitis optica (NMO). In early March, due to lack of response to the high-dose steroid therapy, the patient underwent therapeutic plasmapheresis, followed by intravenous immunoglobulin. Spinal cord MRI showed a reduction of the areas with altered signal intensity [Figure 1b]; nonetheless, there was only a modest clinical improvement. The patient then continued steroid therapy with prednisone at immunosuppressive dosages, and at late March he was transferred to the spinal unit department to proceed with a rehabilitation program.

On July 2013, after 4 months of physiotherapy and steroid treatment, the patient showed full recovery of sphincter control and had recommenced walking with assistance. At that time he still suffered from a deficit in proprioceptive sensibility, as well as pronounced fatigability during movement.

Our examinations ruled out viral myelitis, neurological complications of systemic diseases such as systemic lupus erythematous (SLE), Sjogren’s syndrome and sarcoidosis and atypical presentation of multiple sclerosis (MS). The radiological findings on spinal cord MRI and, later, evidence of AQP4-Ab confirmed the diagnosis of NMO. Devic’s disease is an idiopathic autoimmune syndrome of the CNS characterized by severe attacks of optic neuritis and myelitis. Although the NMO phenotype can occur in contexts of MS or several systemic autoimmune diseases, it is considered a distinct syndrome, especially since the identification of the disease-specific antibody anti-aquaporin 4 (AQP4-Ab, or NMO-Ig) in more than 70% of patients with Devic’s disease [4]. AQP4-Ab is thought to play a critical role in producing the lesions by triggering an immune attack against the aquaporine channel type 4, which is the predominant water channel in the CNS and particularly expressed in the optic nerves and spinal cord [5]. Thus, to our knowledge this is the first description of a severe case of classic NMO (i.e.*,* optic-spinal form) triggered by Peg-IFNα.

We can infer that a prolonged exposure of the patient’s immune system to the immunomodulatory activity of Peg-IFNα could have broken the immune tolerance and started an autoimmune reaction, eventually leading to the clinical manifestations of the neuromyelitis optica. In support of our inference, it has been observed that patients with autoimmune and inflammatory diseases (mainly SLE, but also reumatoid artrithis and MS) possess endogenous IFNα inducers (immune complexes that activate natural IFNα producing cells), which cause continuous IFNα production and subsequent chronic stimulation of the immune system [6,7].

## Conclusions

Worldwide, Peg-IFNα will likely remain a predominant treatment for chronic hepatitis C over the next few years, even though its side effects are sometimes unpredictable and severe. We report this case in order to alert treating physicians about rare but important unexpected complications of interferon. Those kind of life-threatening or long term disabling side effects should be of concern, making it advisable to achieve balance between benefits and risks of IFNα-based regimen, especially in patients not in urgent need of starting therapy, in the hopes that quick development of (and broader access to) new tolerable directing-acting antivirals will make IFNα-free therapy possible in a near future.

## Consent

Written informed consent was obtained from the patient for publication of this Case report and any accompanying images. A copy of the written consent is available for review by the Editor of this journal.

## Abbreviations

HCV: Hepatitis C virus; Peg-IFNα: Pegylated-interferon α; CSF: Cerebrospinal fluid; MRI: Magnetic resonance imaging; CNS: Central nervous system; NMO: Neuromyelitis optica; AQP4-Ab: Antibody anti-aquaporin 4; SLE: Systemic lupus erythematous; MS: Multiple sclerosis.

## Competing interests

Financial competing interests:

This study was supported by Anlaids Sezione Lombarda.

Non-financial competing interests:

The authors declare that they have no competing interest.

## Authors’ contribution

DM wrote the first draft. AS contributed to the manuscript preparation. LB followed the patient in the neurology department and contributed to the manuscript preparation. AB revised the manuscript. CF followed the patient in the Neurology Department and revised the manuscript. AG followed the patient in the Division of Infectious Diseases and revised the manuscript. All authors read and approved the final manuscript.

## Pre-publication history

The pre-publication history for this paper can be accessed here:

http://www.biomedcentral.com/2050-6511/15/56/prepub
